# Large scale hierarchical clustering of protein sequences

**DOI:** 10.1186/1471-2105-6-15

**Published:** 2005-01-22

**Authors:** Antje Krause, Jens Stoye, Martin Vingron

**Affiliations:** 1 Max Planck Institute for Molecular Genetics, Computational Molecular Biology, Ihnestrasse 73, 14195 Berlin, Germany; 2 Universität Bielefeld, Technische Fakultät, AG Genominformatik, Postfach 100131, 33501 Bielefeld, Germany; 3 TFH Wildau, Bahnhofstrasse 1, 15745 Wildau, Germany

## Abstract

**Background:**

Searching a biological sequence database with a query sequence looking for homologues has become a routine operation in computational biology. In spite of the high degree of sophistication of currently available search routines it is still virtually impossible to identify quickly and clearly a group of sequences that a given query sequence belongs to.

**Results:**

We report on our developments in grouping all known protein sequences hierarchically into superfamily and family clusters. Our graph-based algorithms take into account the topology of the sequence space induced by the data itself to construct a biologically meaningful partitioning. We have applied our clustering procedures to a non-redundant set of about 1,000,000 sequences resulting in a hierarchical clustering which is being made available for querying and browsing at .

**Conclusions:**

Comparisons with other widely used clustering methods on various data sets show the abilities and strengths of our clustering methods in producing a biologically meaningful grouping of protein sequences.

## Background

With the overwhelming growth of biological sequence databases, handling of these amounts of data has increasingly become a problem. Protein sequences constitute one such data type. The number of unique entries in all protein sequence databases together exceeds now about a million. However, biological evolution lets proteins fall into so-called families, thus imposing a natural grouping. A protein family contains sequences that are evolutionarily related. Generally, this is reflected by sequence similarity. Therefore, one aims at organizing the set of all protein sequences into clusters based on their sequence similarity.

Clustering a large set of sequences as opposed to dealing only with the individual sequences offers several advantages. A frequent problem is the identification of sequences that are similar to a new query sequence. This task can be executed much quicker when only one comparison to an entire cluster has to be performed rather than one comparison per database sequence. Another application lies in the possibility of analyzing evolutionary relationships among the sequences in a cluster and of the species they come from. Moreover, the presence or absence of sequences of a group of species can give useful information about their evolutionary relationship, if their complete set of protein sequences is known.

The aim of clustering protein sequences is to get a biologically meaningful partitioning. One of the simplest well-studied and computationally cheap methods to construct a clustering of data points is *single linkage clustering*. Starting with the pair of data points of least distance, one incrementally merges single data points or already existing clusters. Such a hierarchical clustering can be viewed as a tree, called the *single linkage tree*. The leaves represent the individual data points, while the root of this tree corresponds to just one large cluster representing the whole data set. All other layers in between can be seen as cluster sets at different levels of similarity. However, it is not clear which layers give a meaningful partitioning of the data. They should be chosen so that they neither produce small trivial clusters nor form huge uninformative clusters.

Several approaches already deal with the problem of partitioning a protein sequence database into protein families. Automatically generated cluster sets like ProtoMap [1], ProtoNet [2], or CluSTr [3] typically provide a hierarchical classification at several different levels of similarity. Others, like iProClass [4] or PIRSF [5] include further knowledge, e.g., from domain based classifications, or require manual interaction. Kawaji *et al*. [6] recently developed a graph-based clustering method for the detection of distantly related sequences of a protein family. TribeMCL [7] is a method for clustering proteins into 'protein families' using a Markov Clustering method. It is primarily used for comparing protein sequence sets of completely sequenced genomes. Reviews of currently available cluster sets can be found in: Heger *et al*. [8] and Liu *et al*. [9].

In our approach we first exploit the branching structure of the single linkage tree, which elucidates an unexpected structuring of the sequence space. Traversing the tree from a leaf towards the root we inspect the sizes of the merging subtrees. First one notices relatively small increases that correspond to very similar proteins. Later on, sequences merging in correspond to weakly related proteins. At one point, however, we observe an enormous increase in the size of the subtree, where a large part of the database merges in. All sequences below this point in the tree are assumed to belong to the same *superfamily*. Each superfamily typically covers several closely related protein families. They can be determined by revealing the connectivity of the sequences belonging to a superfamily. Since the single linkage tree is built using only the smallest distances connecting subtrees, information about the connectivity within these subtrees is lost in the hierarchy. For each superfamily, we construct a *superfamily distance graph *by including only those nodes labeled with sequences belonging to the respective superfamily. These graphs are then split at reasonable cut sites into *highly connected subclusters*. For historical reasons [10], we call our procedure as well as the resulting cluster set SYSTERS, which is short for *SYSTEmatic Re-Searching*.

Up to that point, the hierarchy consists of superfamily and family clusters. However, protein sequences are built up of smaller entities, called *domains*. They again can be grouped independently of a certain order in a protein sequence. For this level we rely on one of the currently established domain databases, i.e., the Pfam database [11]. To allow the user to explore protein sequence space through the complete hierarchy, we present an interface to our cluster set on the Internet. It is possible to enter the hierarchy at each of the layers through various entry points and change to another layer whenever desired. Additional information like a multiple alignment or a phylogenetic tree is given for each of the family clusters.

Here, we explain in more detail the SYSTERS algorithms developed to determine superfamily and family clusters. Each step is illustrated by an example. We report our results on clustering the non-redundant protein sequence space consisting of about 1,000,000 sequences. An overview of the availability and accessibility of the cluster set is given. Finally, we present a comparison of our clustering method with two other currently available and widely used clustering methods, namely single linkage clustering and TribeMCL.

## Results and discussion

### Clustering

We have applied our algorithms as described in the Methods Section to a sequence set consisting of all known protein sequences from the Swiss-Prot Rel. 41 and TrEMBL Rel. 23 databases [12], and from several completely sequenced organisms [13-16]. The original set contains 1,168,542 sequences. Sequences which are too short to yield a result in the database search are removed from this set. Sequences which are identical (sub-)sequences of other sequences are sorted together and only the longest sequence is retained as the representative. In a pairwise comparison of all remaining 969,579 non-redundant sequences, this results in a triangular matrix sparsely filled with 775,133,144 E-values better than or equal to 0.05. Comparisons of a sequence to itself are not considered. By temporarily removing all those sequences which are at least 80% identical over at least 80% of their entire length to another sequence, this number decreases. These sequences are considered redundant, and are added to the cluster set again later in order to retain their annotation. By reducing the number of sequences to 546,538 non-redundant sequences, the remaining number of pairwise comparisons decreases significantly. Fortunately, the resulting triangular distance matrix turns out to be sparsely filled with only 52,618,818 values (0.035% of all possible pairs). Constructing the distance graph with these values, the data splits into 93,918 connected components with 76,347 components consisting of only one sequence. The resulting single linkage tree divides into 147,796 superfamilies with 110,308 of them consisting of only one sequence. The subclustering splits the data further into 158,153 family clusters with an overall number of 110,322 single sequence clusters.

### Access to the cluster set

The SYSTERS cluster set [17] is available over the Internet at . There it is possible to explore the protein sequence space by navigating through the complete hierarchy consisting of superfamilies, family clusters, and domains. For the last layer in the hierarchy, the domain level, we rely on one of the currently established domain databases, namely the Pfam collection of protein domains. It is possible to enter the hierarchy at any layer, e.g., by searching for a keyword, choosing a species, or selecting a domain. For each family cluster a consensus sequence is generated. All consensus sequences together build a database searchable by BLAST. Thus, a clear assignment of a new protein or nucleotide sequence to a family and a superfamily is possible. Additional information like a multiple alignment or a phylogenetic tree is given for each of the family clusters. Whenever possible, links to external resources are provided to allow for further information, e.g., about structural properties or underlying genes.

### Validation

For the validation of our clustering procedures we needed on one hand a "true" biologically verified cluster set and on the other hand results of other clustering procedures on this data set. Unfortunately, for large scale analyses such validated data is not available. Thus, we decided on performing our evaluations on two biologically meanigful data sets, namely well characterized sequences from Swiss-Prot and TrEMBL with (a) Pfam domain annotations and (b) ENZYME annotations.

Clustering of such large data sets is not an every day routine. Normally the software to handle such data sets is not publicly available and only the results of their application are published. Although these results are mostly publicly available for browsing on the web the underlying primary data differs in all of these data sets. Additionally a systematic, unbiased and independent comparison would be intractable on a large scale by querying the web.

One of the simplest well-studied and computationally cheap methods to construct a clustering is single linkage clustering. We implemented procedures to perform a single linkage clustering on the two data sets at various different cutoffs. This corresponds to performing single sequence searches with a certain E-value cut-off for all sequences in the data set with subsequent determination of the connected components of the results. Additionally we decided to compare our clustering procedure to one of the most widely used and publicly available methods for large scale protein sequence clustering, namely TribeMCL.

We applied the single linkage clustering as well as the SYSTERS clustering to the Pfam data set and computed the Jaccard coeffcient, the sensitivity and the selectivity of the clustering results in comparison to the "true" cluster set as described in the Methods Section. All clusterings were performed on the non-redundant data set as described under Preprocessing in the Methods Section. After the clustering, redundant sequences were added again to the cluster sets to allow for a comparison with the "true" cluster sets.

For the Pfam cluster set the best single linkage clustering with respect to the "true" cluster set can be achieved at an E-value cutoff of 1e-53 (cf. Table 1). The SYSTERS clustering results in a slightly higher Jaccard value. Note that the "best" single linkage clustering result can not be determined from the clustering itself, but was selected after comparison with the "true" cluster set, which is not available when clustering new sequence data. Thus, the SYSTERS clustering turns out to be superior to the single linkage clustering in the sense that it is able to determine the correct cluster granularity without manual interaction.

**Figure 1:**
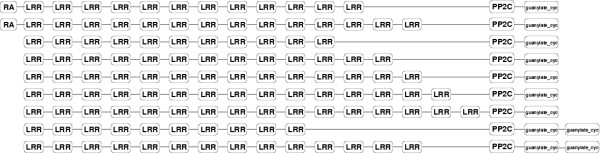
**Multi domain proteins **Sequences with different domain compositions belong to the same family of Adenylate cyclases but form different "true" clusters (Pfam domains: RA: Ras association (RalGDS/AF-6) domain; LRR: Leucine Rich Repeats; PP2C: Protein phosphatase 2C; guanylate_cyc: Adenylate and Guanylate cyclase catalytic domain)

In total we get only weak results for the Pfam data set. One of the reasons is the choice of the "true" cluster set. Figure 1 shows an example where sequences composed of the same domains and belonging to the same family of adenylate cyclases end up in different "true" clusters. The repetition of one domain and the presence/absence of another domain lets them fall into different "true" clusters. These sequences are in a biological sense correctly clustered by SYSTERS but cause a problem when comparing them to the "true" cluster set. In this case the "true" clusters build subsets of the SYSTERS subclusters.

Another reason for the weak results in comparison with the Pfam data set are fusion proteins. They bring together sequences belonging to otherwise unrelated families.

We applied the single linkage clustering, the SYSTERS clustering and the TribeMCL clustering to the ENZYME data sets and computed the Jaccard coeffcient, the sensitivity and the selectivity of the clustering results in comparison to the "true" cluster sets as described in the Methods Section. For this data set the SYSTERS clustering turns out to be superior to both the single linkage clustering and TribeMCL (cf. Table 1). In both ENZYME data sets the TribeMCL clustering shows the best ability to reject unrelated sequences but at the expense of finding distantly related sequences. As expected, the SYSTERS subclustering shows the best result on the lowest level of the ENZYME data set where individual enzymes are identified.

In total all methods perform significantly better on the ENZYME data set. This data set is much smaller than the Pfam data set and contains well annotated enzymes. In contrast to the Pfam data set, the "true" cluster set was chosen on the basis of enzyme annotation, namely EC numbers, as described in the Methods section. Sequences belonging to the same "true" cluster thus may show the same domain composition but may also differ in this sense. Although this is a somehow weaker definition of a "true" cluster set it is more focussing on the functional properties of the proteins.

## Conclusions

We have presented a hierarchical clustering of protein sequences into biologically meaningful superfamily and family clusters. A combination of an upward sweep with dynamic determination of superfamily cutoffs and a downward pass that divides superfamilies into families has been introduced. We determine a superfamily by detecting the largest increase in the size of the merging subtree traversing from a leaf in a single linkage tree to the root. We assume that at this point the twilight zone begins because suddenly a large number of supposedly unrelated sequences enters the cluster. Each of the superfamilies is further cut into family clusters by detecting weak connections in the underlying distance graph.

It is interesting that both the superfamilies as well as the family clusters are generated solely from the structure of the single linkage tree (respectively the underlying distance graph), without any knowledge of the biological information represented. Such self-structuring properties have also been observed in other large data sets such as phone-call or web-link graphs [18].

An alternative approach for cluster determination is presented by Sharan *et al*. [19]. Their CLICK algorithm (Cluster Identification via Connectivity Kernels) uses graph-theoretic and statistical techniques to first identify tight groups of highly similar elements (kernels), which are likely to belong to the same cluster. Several heuristic procedures are then used to expand the kernels into the full clustering. In our much simpler approach, we produce a hierarchical clustering based on the partitioning into superfamilies, which already results in a biologically meaningful set of family clusters.

Although the vast majority of cases we looked at are in agreement with biological knowledge, there exist some inconsistencies due to peculiarities in the data. Distinct protein families may end up erroneously in the same superfamily because of a fusion protein covering sequence information from both families. The same effect can be seen at multidomain protein families linked together by a single highly conserved common domain. Although the subclustering in most cases splits these superfamilies again into distinct families, we would prefer to take care of these cases already in the process of superfamily determination. Nevertheless, comparisons with other methods showed that our clustering methods are able to produce a biologically meaningful clustering.

Thus far, our hierarchy consists of two layers representing protein superfamilies and families. For the third layer located at the domain level, we currently rely on well-established domain databases, but intend to follow our methodology also in the direction of deriving so far unknown domains.

Future plans also include a regular update of the SYSTERS cluster set. Since the most time consuming part are the all-against-all sequence searches, new sequence similarities will be incrementally added instead of recalculating all similarity values. The clustering procedures themselves rely on the topology of the whole sequence space and can be run on the whole data set whenever the underlying sequence set changes. Other future developments will be in the direction of the so called *tree of life*. We plan to combine the evolutionary information given by each of the protein clusters to extend the knowledge about the relationship between different groups of species.

## Methods

### Clustering procedures

**Figure 2:**
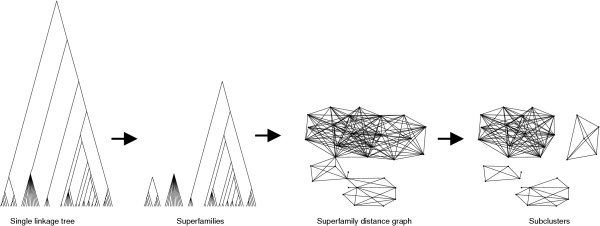
**Schematic overview of the clustering procedures **We start with a single linkage tree constructed from pairwise distances. Each leaf in the tree corresponds to a protein sequence. Superfamilies are determined based on the internal structure of the tree. For each superfamily a distinct superfamily distance graph is built. This weighted graph is cut at weak connections into subclusters.

Here we present the methods that we use to compute our clustering of protein sequences, i.e., selecting superfamilies and dividing them into reasonable family clusters. Figure 2 shows a schematic overview.

#### Preprocessing

The total number of entries in all protein sequence databases together now exceeds about a million. This number includes fragmental as well as identical (sub-)sequences from different resources. To reduce the amount of data without losing information we exclude redundant information in the form of identical and nearly identical (sub-)sequences from the data set prior to the clustering.

We model the remaining protein space as a weighted undirected graph with pairwise distances attached to the edges. We decided on using E-values computed from pairwise local sequence alignments [20] as distances (all-against-all database searches were carried out on a Paracel GeneMatcher™ machine [21]). The E-value (short for Expectation value) is the number of alignments with similarity scores equivalent to or better than the score *S *that one expects to find in a database search by chance. Thus, the lower the E-value, the more significant is the score. Typically, matches with an E-value lower than 1e-20 are assumed to be relevant, while those sequence pairs with an E-value higher than 0.01 need further experimental evidence to be accepted as being distantly related. Values in between belong to the so called *twilight zone*, and a clear statement about relatedness cannot be made for them. All sequence pairs whose E-value was worse than 0.05 were assumed to be unrelated and their distance was set to infinity. We are aware that we may miss distantly related sequences with this E-value threshold in a single sequence search. However, by using each sequence in the data set as query in a database search and combining all results we hope to overcome this problem. The resulting symmetric distance matrix *D *contains all pairwise distances *d*(*s*_*i*_, *s*_*j*_) for each pair of protein sequences *s*_*i *_and *s*_*j*_, 1 ≤ *i*, *j *≤ *n*, for which *d*(*s*_*i*_, *s*_*j*_) < ∞.

#### Single linkage tree

The distance matrix *D *can be represented by an undirected weighted graph *G*, which we call the *distance graph*. *G *= (*V*, *E*) is defined as follows: *V *= {*v*_*i *_| *v*_*i *_= {*s*_*i*_}, *i *∈ {1, ..., *n*}} and *E *= {(*v*_*i*_, *v*_*j*_) | *i*, *j*, ∈ {1, ..., *n*}, *i *≠ *j*, *d*(*s*_*i*_, *s*_*j*_) < ∞}. The weight *w*(*v*_*i*_, *v*_*j*_) of an edge (*v*_*i*_, *v*_*j*_) ∈ *E *is given by *w*(*v*_*i*_, *v*_*j*_) = *d*(*s*_*i*_, *s*_*j*_).

The single linkage tree is built based on the distance graph *G *in an agglomerative manner. The algorithm starts with a forest (collection of trees) *F *where each sequence corresponds to a distinct tree. As long as there are edges in the graph *G*, the edge with the smallest weight is selected and the adjacent nodes in *G *are merged. Edges linking this newly created node to adjacent ones in the graph receive the weight of the smaller of the two original edges. The two corresponding trees in *F *are collected together in a new tree rooted by a parental node labeled with the connecting edge weight. Finally, to allow for a better handling of the data, the resulting unconnected trees are rooted by connecting their roots to an artificial overall root node with weight infinity.

#### Superfamily determination

Different protein superfamilies display a different degree of conservation. Therefore, for each superfamily, the twilight zone starts at a different cutoff. A crucial problem thus lies in the determination of an appropriate E-value threshold for each superfamily. To this end we have devised the following procedure. For an edge of the tree linking, say, a parent *p *and a child *q*, we compute the quantity


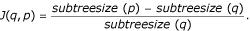


**Figure 3:**
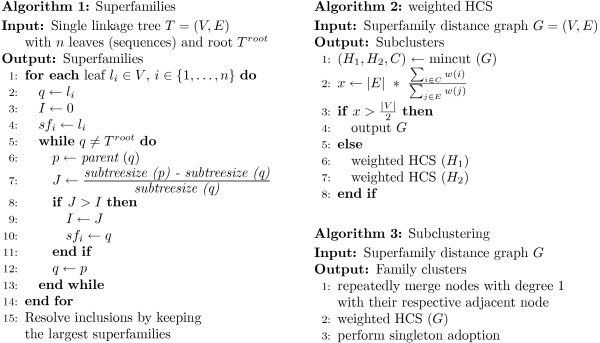
The SYSTERS algorithms

**Figure 4:**
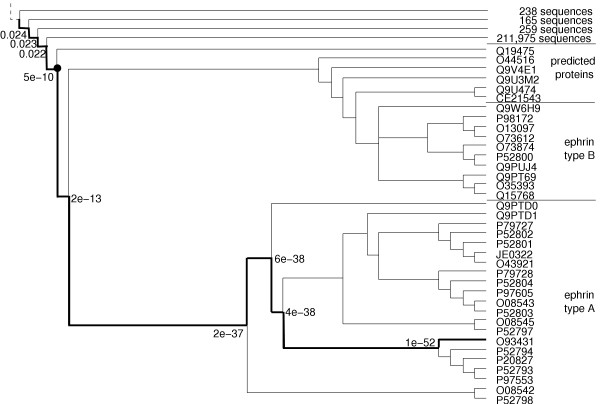
**Excerpt from the single linkage tree **The superfamily of sequence O93431 is determined as follows (traversing the tree along the branches depicted as bold lines). The first internal node connects this sequence with the four sequences P52794, P20827, P52793, and P97553 at an E-value of 1e-52. Thus, the ratio of the size of the merging subtree and the size of the current subtree at this point is 4/1. Stepping up the hierarchy, the next node (E-value 4e-38) connects these five sequences with a subtree consisting of 13 sequences, resulting in a ratio of 13/5 (= 2.6). Stepping further up the hierarchy, the following ratios are 1/18 (= 0.056 at E-value 6e-38), 2/19 (= 0.105 at E-value 2e-37), 15/21 (= 0.714 at E-value 2e-13), 1/36 (= 0.028 at E-value 5e-10), 211 975/37 (= 5729.054 at E-value 0.022), 259/212 012 (= 0.001 at E-value 0.023), etc. Taking the maximum of the ratios we find the superfamily root at E-value 5e-10 as the last node before the largest relative increase (depicted as a bullet in the tree). The superfamily of sequence O93431 hence consists of the 37 sequences belonging to the ephrin type A and type B families plus a few predicted proteins.

*J *represents the ratio between the size of all the subtrees below *p *without the child *q *and the size of the subtree below *q*. Watching the development of *J *as one walks up the tree from a leaf towards the root, one can observe that *J *tends to increase dramatically as one leaves the superfamily to which the leaf belongs, and then decreases again. This intuition is captured by our algorithm. For each leaf, we determine the maximum *J *as one proceeds from the leaf to the root of the single linkage tree. This strategy is applied to all leaves in the tree, assigning a superfamily to each leaf. In the end, inclusions are resolved by keeping the largest superfamilies. We call the internal node induced by a superfamily the *superfamily root*. The E-value linked to this node is called the *superfamily cutoff*. Refer to Algorithm 1 in Figure 3 for more details. Figure 4 shows an example of the superfamily determination. Only a part of the complete single linkage tree consisting of 290,811 leaves and 186,176 internal nodes is shown. The superfamily procedure correctly determines the ephrin family of sequences. Ephrins are membrane-attached proteins involved in the development of the nervous system and can be further distinguished into type A and type B ephrins depending on their membrane binding mechanism.

#### Subclustering

Stepping down the hierarchy of the single linkage tree starting at a superfamily root usually splits off one sequence after another, but does not lead to a meaningful partitioning into families. Since the single linkage tree is built using only the best (lowest) E-values connecting subtrees, information about the connectivity within these subtrees is lost in the hierarchy. For each superfamily we construct a distance graph that includes only those nodes labeled with sequences belonging to the respective superfamily and those of the induced edges which are labeled with a distance better than or equal to the superfamily cutoff. Let *SF *be the set of sequences belonging to the superfamily *sf *and *c *the corresponding superfamily cutoff. We call the connected weighted graph *G *= (*V*, *E*) with *V *= {*v*_*i *_| *v*_*i *_= {*s*_*i*_}, *s*_*i *_∈ *SF*} and *E *= {(*v*_*i*_, *v*_*j*_) | *w*(*v*_*i*_, *v*_*j*_) = *d*(*s*_*i*_, *s*_*j*_), *s*_*i*_, *s*_*j *_∈ *SF*, *i *≠ *j*, *d*(*s*_*i*_, *s*_*j*_) ≤ *c*} the *superfamily distance graph *of *sf*.

To split a superfamily distance graph into family clusters, we use an algorithm that can be seen as a weighted version of a method presented by Hartuv *et al*. [22]. First, we review some standard graph-theoretic definitions. The *edge-connectivity k*(*G*) of a graph *G *is the minimum number *k *of edges whose removal results in a disconnected graph. A *cut *in a graph is a set of edges *C *whose removal disconnects the graph into two disjoint components *H*_1 _and *H*_2_. A *minimal cut *is a cut with a minimum number of edges. The length *p*(*u*, *v*) of the *shortest path *between nodes *u *and *v *in *G *is the minimum length of a path from *u *to *v*, if such a path exists; otherwise *p*(*u*, *v*) = ∞. The *diameter *of a connected graph *G *is the maximum shortest path length *p*(*u*, *v*) over all pairs of nodes *u *and *v *in *G*.

The key definition of the algorithm in [22] is the following: An undirected unweighted graph *G *with *n *> 1 nodes is called *highly connected*, if *k*(*G*) >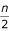. A *highly connected subgraph *(HCS) is an induced subgraph *H *⊆ *G*, such that *H *is highly connected. In an unweighted graph this definition results in the following property: The diameter of every highly connected subgraph is at most two. Thus, these subgraphs are compact clusters which need not meet the constraint of being fully connected.

The original HCS algorithm in [22] recursively splits a connected graph at a minimal cut site until a disjoint set of *highly connected subclusters *is reached. For our purposes we had to modify the algorithm to be able to handle a weighted graph. Precisely, in our weighted HCS algorithm, if the edge weights covered by the minimal cut are approximately the same as in the remaining graph, the graph is assumed to be already highly connected and is not further split into subclusters (see Algorithm 2 in Figure 3).

The E-values in our data set range from 0 (corresponding to any E-value better than 1e-180) to 0.05. To be able to find a minimal cut in our graph, edge labels should be positive values with a low value representing a weak connection and a high value representing a strong connection. Instead of using the raw E-values we label the edges in our graph with the negative logarithm of the corresponding E-value each. Since the logarithm of 0 is not defined, we use an arbitrary value (e.g., 181) for these edges instead of the logarithm. The running time of both HCS algorithms is bounded by 2N * *f*(*n*, *m*), where *N *is the number of clusters found and *f*(*n*, *m*) is the time complexity of computing a minimum cut in a graph with *n *nodes and *m *edges. We use the implementation of the "mincut" algorithm given in the LEDA [23] distribution, which has a time complexity of 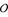
(*nm *+ *n*^2 ^log *n*).

To apply this algorithm to our data set we added a preprocessing as well as a postprocessing step as shown in Algorithm 3 in Figure 3. First, we describe the preprocessing. Cuts consisting of only one edge in the graph will be found first by the mincut algorithm, but are as time consuming to find as other cuts.

Sequences being connected with the remaining graph by only one edge are either fragmental or are the so far sole representative of a protein family in the sequence database. The underlying data of our clustering is known to contain lots of fragmental sequences. Before applying the HCS algorithm to our graph, we repeatedly merge all nodes connected to the remaining graph with only one edge with their respective adjacent node.

Nevertheless, the HCS algorithm may split off single sequences as subclusters. Thus, in a postprocessing step, sequences which ended up after the subclustering as a single sequence cluster are assigned to their closest neighboring cluster (*singleton adoption*), if there is no contradiction. When there are several minimum cuts in a graph, the original as well as our weighted HCS algorithm might choose a minimum cut which, from the clustering point of view, is not optimal. In many cases this process will break clusters into singletons. In the original algorithm in [22] iterations were introduced to handle these cases. Since we are working on a weighted graph these cases occur very rarely and mostly are compensated by the subsequent singleton adoption step.

**Figure 5:**
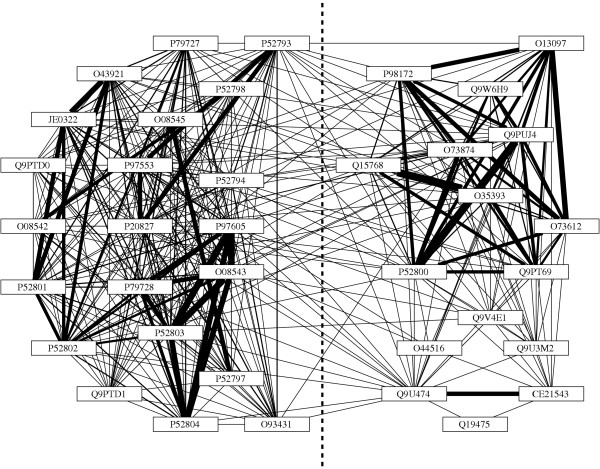
**The superfamily distance graph of the ephrin superfamily **The graph contains only those edges which represent E-values of at least the superfamily cutoff 5e-10. The width of an edge is according to its E-value, here ranging from 5e-10 (thinnest edge) to 3e-149 (thickest edge). The subclustering procedure first splits off nodes from the bottom right of the graph as single sequence clusters. These sequences are predicted proteins which are not yet confirmed as functioning by any experiment. The last accepted split in the graph results in the partitioning into the two major groups of ephrin type A (left) and type B (right) sequences as shown by the dashed line. Single sequence clusters are added to the ephrin type B family in the subsequent singleton adoption step.

Figure 5 shows an example of splitting the superfamily distance graph of the ephrin superfamily (see Fig. 4) into two subclusters representing ephrin types A and B.

### Validation

#### Pfam sequence set

For our analyses we used all sequences from the Swiss-Prot and TrEMBL databases annotated with Pfam domains (Rel. 9). This data set consists of 5,724 single domain families assigned to 733,830 sequences. Since our aim is not to cluster single domains but full-length sequences, we define a "true" cluster consisting of all sequences having the same domain composition. Fragmental sequences will cause a problem in our analyses by showing a different domain composition than complete sequences. We restrict our analyses to sequences not annotated as being fragmental in the Swiss-Prot or TrEMBL databases. The resulting "true" cluster set thus consists of 442,872 sequences sorted into 16,990 distinct families.

#### ENZYME sequence set

The ENZYME database [24] stores data of a functional classification system based on function rather than sequence or structure. Each enzyme of known function is given an EC (Enzyme Commission) Number of the form A.B.C.D with

A : type of reaction catalyzed (at present 6 classes)

B : subclass, information about type of compound or group involved

C : sub-subclass, further specifies the nature of the reaction

D : serial number to identify individual enzyme within sub-subclass

Although several distinct proteins may catalyze the same reaction, they are all ascribed the same EC number, since the naming system is based upon the reaction catalyzed. Thus, sequences given the same EC number do not necessarily show sequence similarity.

For our analyses we used all sequences from the Swiss-Prot and TrEMBL databases annotated with a unique EC number. We define two different "true" cluster sets representing different levels of granularity as follow: (1) sequences having A, B, C, and D in common build a cluster, (2) sequences having A, B, and C in common build a cluster. The data set consists of 84,405 sequences.

#### Clustering coeffcient

Assuming we have a well defined cluster set, we can compare our cluster set with this "true" cluster set based on the following numbers:

Number of sequence pairs clustered together in

*a*: both cluster sets ("true positives").

*b*: the "true" cluster set, but not in our cluster set ("false negatives").

*c*: our cluster set, but not in the "true" cluster set ("false positives").

As similarity measure we decided on the Jaccard similarity [25] defined as follows: 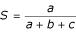. A perfect clustering which is identical to the "true" cluster set would result in *S *= 1.

Additionally we calculated the sensitivity (the ability to detect distantly related sequences: 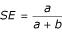) and the specificity (the ability to reject non-related sequences: 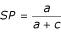) for all cluster sets.

#### Single linkage clustering

We performed a single linkage clustering at various static E-values from 1e-02 to 1e-180. All resulting cluster sets have in common that when plotting the number of clusters against the cutoff E-value, one observes a continuous, smooth curve, indicating that there is no obvious (biologically given) choice of a cutoff (data not shown).

#### TribeMCL

TribeMCL [7] is a method for clustering proteins into 'protein families' using a Markov Clustering method. It is primarily used for comparing protein sequence sets of completely sequenced genomes. We performed TribeMCL clustering (Version 03–276) with different inflation value settings ranging from 1.1 to 5 for all data sets. The inflation parameter is part of the core MCL algorithm and influences the granularity (or size) of the output clusters. For very small or 'tight' protein families an inflation value setting of 4.0 or 5.0 is recommended. For larger (broader) protein families settings of 1.1, 2.0 and 3.0 can be used.

For the Pfam data set we were not able to perform TribeMCL clustering due to memory allocation problems while executing the program.

## Authors' contributions

MV had the initial ideas for SYSTERS. JS developed the superfamily determination. AK developed the subfamily determination, implemented the workflow from the raw sequence databases to a working web-server and made the validations. All authors read and approved the final manuscript.

## Figures and Tables

**Table 1:** Comparison of SYSTERS, TribeMCL and single linkage clustering (SLC) on Pfam and ENZYME data sets (J: Jaccard Coeffcient, SE: Sensitivity, SP: Specificity). The best result in each row is shown in bold face. For the single linkage clustering only the results of the "best" clustering are shown together with the corresponding cutoff E-value. In the case of Sensitivity/Specificity these values were choosen according to the intercept point of the two curves when plotting the values for all possible E-value cutoffs. All clustering procedures were applied to the non-redundant data set and redundant sequences were added to the cluster sets again to compare to the "true" cluster sets: ^*a*^33,963,365 pairwise values of 283,113 non-redundant sequences used for clustering and 442,872 redundant sequences used in comparison; ^*b*^1,582,948 pairwise values of 38,176 non-redundant sequences used for clustering and 84,405 redundant sequences used in comparison.

	SLC		SYSTERS		TribeMCL at Inflation				
	best	at cutoff	Superfam.	Subclust.	1.1	2	3	4	5
Pfam^*a*^									

J	0.19362	1e-53	0.15637	**0.20815**	---	---	---	---	---
SE	0.26886	1e-49	**0.55272**	0.48302	---	---	---	---	---
SP	0.26536	1e-49	0.17902	**0.26781**	---	---	---	---	---

ENZYME^*b*^									

A.B.C.D									

J	0.88760	1e-21	0.77445	**0.89670**	0.60390	0.60074	0.59990	0.59942	0.59778
SE	0.92295	1e-08	**0.92931**	0.92297	0.61323	0.60328	0.60224	0.60164	0.59989
SP	0.93616	1e-08	0.82294	0.96924	0.97543	0.99304	0.99357	0.99388	**0.99416**

A.B.C.?									

J	0.71527	1e-15	0.65915	**0.72410**	0.48721	0.47900	0.47803	0.47746	0.47600
SE	0.74985	1e-03	**0.75320**	0.73727	0.49099	0.47996	0.47895	0.47836	0.47688
SP	0.80855	1e-03	0.84073	0.97592	0.98445	0.99586	0.99601	0.99608	**0.99617**
